# Differential gene expression in human abdominal aortic aneurysm and aortic occlusive disease

**DOI:** 10.18632/oncotarget.3848

**Published:** 2015-04-15

**Authors:** Erik Biros, Gabor Gäbel, Corey S. Moran, Charlotte Schreurs, Jan H. N. Lindeman, Philip J. Walker, Maria Nataatmadja, Malcolm West, Lesca M. Holdt, Irene Hinterseher, Christian Pilarsky, Jonathan Golledge

**Affiliations:** ^1^ The Queensland Research Centre for Peripheral Vascular Disease, College of Medicine and Dentistry, James Cook University, Townsville, Queensland, Australia; ^2^ Department of Vascular and Endovascular Surgery, Ludwig-Maximillian University, Munich, Germany; ^3^ Department of Vascular Surgery, Leiden University Medical Center, Leiden, The Netherlands; ^4^ Royal Brisbane Clinical School, The University of Queensland, Queensland, Australia; ^5^ The Cardiovascular Research Group, Department of Medicine, The University of Queensland, Queensland, Australia; ^6^ Institute of Laboratory Medicine, Ludwig Maximilians University Munich, Munich, Germany; ^7^ Department of General, Visceral, Vascular and Thoracic Surgery, Charité Universitätsmedizin Berlin, Charité Campus Mitte, Berlin, Germany; ^8^ Department of Vascular, Thoracic and Visceral Surgery, TU-Dresden, Dresden, Germany; ^9^ Department of Vascular and Endovascular Surgery, The Townsville Hospital, Townsville, Queensland, Australia

**Keywords:** aortic aneurysm, abdominal, arterial occlusive diseases, tissue array analysis

## Abstract

Abdominal aortic aneurysm (AAA) and aortic occlusive disease (AOD) represent common causes of morbidity and mortality in elderly populations which were previously believed to have common aetiologies. The aim of this study was to assess the gene expression in human AAA and AOD. We performed microarrays using aortic specimen obtained from 20 patients with small AAAs (≤ 55mm), 29 patients with large AAAs (> 55mm), 9 AOD patients, and 10 control aortic specimens obtained from organ donors. Some differentially expressed genes were validated by quantitative-PCR (qRT-PCR)/immunohistochemistry. We identified 840 and 1,014 differentially expressed genes in small and large AAAs, respectively. Immune-related pathways including cytokine-cytokine receptor interaction and T-cell-receptor signalling were upregulated in both small and large AAAs. Examples of validated genes included CTLA4 (2.01-fold upregulated in small AAA, *P* = 0.002), NKTR (2.37-and 2.66-fold upregulated in small and large AAA with *P* = 0.041 and *P* = 0.015, respectively), and CD8A (2.57-fold upregulated in large AAA, *P* = 0.004). 1,765 differentially expressed genes were identified in AOD. Pathways upregulated in AOD included metabolic and oxidative phosphorylation categories. The UCP2 gene was downregulated in AOD (3.73-fold downregulated, validated *P* = 0.017). In conclusion, the AAA and AOD transcriptomes were very different suggesting that AAA and AOD have distinct pathogenic mechanisms.

## INTRODUCTION

Peripheral arterial diseases include a collection of occlusive and aneurysmal diseases affecting arteries outside the heart estimated to affect approximately 5% of adults [1]. The mechanisms responsible for ischemic complications of peripheral artery atherosclerosis are believed to be similar to those implicated in coronary atherosclerosis [2]. In keeping with the perceived similar pathogenesis of coronary and peripheral artery disease most of the drug therapies used in patients with these conditions are the same [3]. The mechanisms involved in abdominal aortic aneurysm (AAA) development and their relationship with atherosclerosis are however controversial [4]. Examination of human AAA biopsies has consistently identified concurrent intimal atherosclerosis and most AAAs have been considered to result from atherosclerosis [5, 6]. Some of the risk factors for atherosclerosis and AAA are however distinct, for example diabetes an important positive risk factor for occlusive artery disease is a negative risk factor for AAA [4, 5]. Dyslipidemia is also believed to be a less important risk factor for AAA than atherosclerotic occlusive disease [7]. There are also a number of similarities between atherosclerosis and AAA, including common risk factors of smoking, hypertension, and male gender [5, 8, 9]. The role of atherosclerosis in AAA therefore is currently controversial [4].

Examination of mechanisms involved in AAA has mainly relied on use of animal, particularly rodent, models [10]. The availability of whole genome expression profiling has allowed insight into a range of human pathologies but has been relatively sparingly applied to AAA and aortic atherosclerosis [11-15]. Two previous studies have examined the whole genome expression profile of human AAA specimen although sample sizes for these studies were relatively small totaling 7 and 10 AAA patients, and no comparison was performed with atherosclerosis [11, 12]. In the current study we examined the whole genome expression profiles of a relatively large number of human aortic biopsies from subjects undergoing surgery to repair AAAs and to treat chronic lower limb ischemic due to aortic atherosclerosis (aortic occlusive disease, AOD). Aortic wall samples obtained from organ donors were used as controls. We aimed to compare the aortic gene expression profile of patients with AAA and AOD to those of older subjects with normal aortic histology.

## RESULTS

### Patient characteristics

The gene expression was initially assessed in the aortic biopsies of 20 patients with small AAAs (mean AAA diameter 53.4±2.3 mm), 29 patients with large AAAs (mean AAA diameter 68.4±14.3 mm), 9 patients with AOD (mean aortic diameter 19.6±2.6 mm), and 10 control organ donors using microarrays (Table 1). AAA patients were less likely to be female compared to controls (small AAA 0%, large AAA 7%, controls 40%; *P* < 0.05), while AOD patients were younger compared to controls (mean age 61.6±9.3 *vs*. 68.4±4.5 years, *P* < 0.05). The validity of microarray findings was further assessed in biopsies obtained from 6 patients with small AAAs, 9 patients with large AAAs, 8 AOD patients, and 6 controls (Table 1). AAA and AOD cases were older compared to controls (mean age small AAAs 69.7±3.8, large AAAs 71.5±7.8, AOD 67.8±4.2, controls 42.7±12.5 years; *P* < 0.05). Mean maximum AAA diameter was 48.5±5.7 and 65.9±7.3 mm in patients with small and large AAAs, respectively.

**Table 1:** Characteristics of subjects included in this study

Characteristic	Discovery group				Validation group			
	Small AAA	Large AAA	AOD	Controls	Small AAA	Large AAA	AOD	Controls
Number	20	29	9	10	6	9	8	6
Aortic diameter (mm)	53.4±2.3	68.4±14.3	19.6±2.6	25.7±1.2	48.5±5.7	65.9±7.3	-	-
Women	*0 (0%)	*2 (7%)	1 (11%)	4 (40%)	1 (17%)	2 (22%)	2 (25%)	2 (33%)
Age (years)	68.8±6.9	70.5±7.1	*61.6±9.3	68.4±4.5	*69.7±3.8	*71.5±7.8	*67.8±4.2	42.7±12.5
PAD	3 (15%)	5 (17%)	9 (100%)	-	0 (0%)	0 (0%)	8 (100%)	-
Hypertension	15 (75%)	25 (86%)	8 (89%)	-	4 (67%)	6 (67%)	7 (88%)	-
Diabetes mellitus	4 (20%)	7 (24%)	1 (11%)	-	4 (67%)	1 (11%)	1 (13%)	-
Dyslipidemia	15 (75%)	20 (69%)	8 (89%)	-	6 (100%)	5 (56%)	6 (75%)	-
Coronary heart disease	8 (40 %)	17 (59%)	7 (78%)	-	4 (67%)	5 (56%)	4 (50%)	-
Ever smoker	7 (35%)	21 (72%)	9 (100%)	5 (50%)	4 (67%)	6 (67%)	8 (100%)	-
BMI (kg/m^2^)	28.8±3.2	26.8±4.0	23.7±2.4	22.7±9.6	34.0±5.5	25.3±3.6	21.9±4.1	-

AAA, abdominal aortic aneurysm; AOD, aortic occlusive disease; BMI, body mass index. Nominal variables are presented as numbers; continuous variables are presented as mean ± standard deviation. AAA or AOD were compared with controls. Continuous variables were compared using Mann Whitney U test; nominal variables were compared using Fisher's exact test

* Asterisk indicates statistically significant differences compared to the control group (*P* < 0.05).

### Numerical assessment of gene expression profiles in small AAAs, large AAAs, and AOD

**Figure 1:**
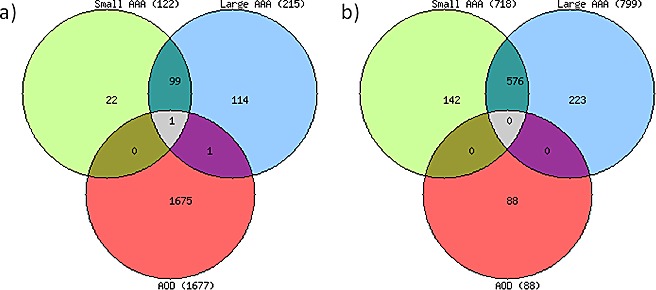
Distribution of differentially expressed genes in aortic aneurysmal and occlusive disease Venn diagrams depict the overlap among differentially expressed genes in patients with small and large abdominal aortic aneurysm (AAA) and aortic occlusive disease (AOD). Shown are a number of upregulated (**a**) and downregulated (**b**) genes. Patients with small or large AAA or AOD were compared with patients without AAA (Controls), (> 2-fold difference, *P* value < 0.05 calculated with non-parametric Mann-Whitney test and corrected by Benjamini and Hochberg method).

A total of 39,157 transcripts in small AAAs, 39,389 transcripts in large AAAs, 37,904 transcripts in AOD, and 36,725 transcripts in control samples, representing > 80% of the Illumina HumanHT-12 v4 Expression BeadChip reference list, were expressed above background and subjected to differential gene expression analysis. A total of 840 individual genes were differentially expressed (≥ 2.0-fold, adjusted *P* < 0.05) in small AAAs compared to controls (122 upregulated and 718 downregulated genes; Figure 1a and 1b, respectively). A total of 1,014 individual genes were differentially expressed (≥ 2.0 fold, adjusted *P* < 0.05) in large AAAs compared to controls (215 upregulated and 799 downregulated genes; Figure 1a and 1b, respectively). 1,765 individual genes were differentially expressed (≥ 2.0-fold, adjusted *P* < 0.05) in AOD compare to controls (1,677 upregulated and 88 downregulated genes; Figure 1a and 1b, respectively). Full lists of differentially expressed genes are given in Supplemental Table I, Supplemental Table II, and Supplemental Table III for small AAA, large AAA, and AOD, respectively. A preponderance of genes upregulated in small AAAs (100/122; 82%) were also upregulated in large AAAs. Approximately 47% (100/215; Figure 1a) of the genes upregulated in large AAAs were also upregulated in small AAAs. In contrast only one gene (*CXCL12*) differentially expressed in AOD samples (1/1,765) was also differentially expressed in small (1/840) or large (1/1,014) AAAs (Figure 1a and 1b).

### Pathways related to the differentially expressed genes in small and large AAA

We performed an enrichment analysis of differentially expressed genes with particular attention to Kyoto Encyclopedia of Genes and Genomes (KEGG) pathways to assess the biological contexts of these genes. KEGG annotations included 93% (114/122) of upregulated genes and 92% (661/718) of downregulated genes in small AAAs; and 91% (196/215) of upregulated genes and 95% (756/799) of downregulated genes in large AAA samples. The KEGG analysis revealed marked upregulation of genes related to immune responses in both small and large AAA. These pathways included cytokine-cytokine receptor interaction (hsa04060; *P* = 1.15*10^−14^ for small AAA, and *P* = 2.52*10^−17^ for large AAA), chemokine signaling pathway (hsa04062; *P* = 4.50*10^−11^ for small AAA, and *P* = 3.53*10^−12^ for large AAA), and T-cell receptor signaling pathway (hsa04660; *P* = 4.07*10^−05^ for small AAA, and *P* = 2.40*10^−06^ for large AAA) as shown in Table 2. Metabolic pathways (hsa01100) and oxidative phosphorylation (hsa00190) were found among the top 10 most significant KEGG categories enriched by downregulated genes with *P* = 2.30*10^−22^ (metabolic pathways) and *P* = 4.25*10^−20^ (oxidative phosphorylation) for small AAA; and *P* = 1.05*10^−24^ (metabolic pathways) and *P* = 8.60*10^−20^ (oxidative phosphorylation) for large AAA (Table 2).

**Table 2:** Top 10 KEGG pathways enriched in small and large abdominal aortic aneurysm

Category	Pathway	KEGG	N	n	P value
Small AAA-upregulated genes	Cytokine-cytokine receptor interaction	hsa04060	267	15	1.15*10^−14^
	Chemokine signaling pathway	hsa04062	190	11	4.50*10^−11^
	Hematopoietic cell lineage	hsa04640	88	7	3.68*10^−08^
	NOD-like receptor signaling pathway	hsa04621	62	6	1.18*10^−07^
	Cell adhesion molecules (CAMs)	hsa04514	134	7	4.15*10^−07^
	Jak-STAT signaling pathway	hsa04630	155	7	9.36*10^−07^
	Intestinal immune network for IgA production	hsa04672	50	4	4.07*10^−05^
	T cell receptor signaling pathway	hsa04660	108	5	4.07*10^−05^
	Cytosolic DNA-sensing pathway	hsa04623	56	4	5.46*10^−05^
	Pathways in cancer	hsa05200	330	7	8.39*10^−05^
Small AAA-downregulated genes	Ribosome	hsa03010	88	27	2.50*10^−26^
	Metabolic pathways	hsa01100	1104	69	2.30*10^−22^
	Oxidative phosphorylation	hsa00190	135	26	4.25*10^−20^
	Parkinson's disease	hsa05012	133	25	3.60*10^−19^
	Alzheimer's disease	hsa05010	169	27	6.00*10^−19^
	Huntington's disease	hsa05016	185	28	6.00*10^−19^
	Focal adhesion	hsa04510	201	23	5.48*10^−13^
	Cardiac muscle contraction	hsa04260	79	15	7.66*10^−12^
	Arrhythmogenic right ventricular cardiomyopathy	hsa05412	76	11	1.65*10^−07^
	Vascular smooth muscle contraction	hsa04270	115	13	1.65*10^−07^
Large AAA-upregulated genes	Cytokine-cytokine receptor interaction	hsa04060	267	20	2.52*10^−17^
	Chemokine signaling pathway	hsa04062	190	14	3.53*10^−12^
	Hematopoietic cell lineage	hsa04640	88	10	9.95*10^−11^
	Primary immunodeficiency	hsa05340	35	7	1.89*10^−09^
	Cell adhesion molecules (CAMs)	hsa04514	134	9	7.87*10^−08^
	Intestinal immune network for IgA production	hsa04672	50	6	6.70*10^−07^
	Cytosolic DNA-sensing pathway	hsa04623	56	6	1.15*10^−06^
	NOD-like receptor signaling pathway	hsa04621	62	6	1.86*10^−06^
	Jak-STAT signaling pathway	hsa04630	155	8	2.30*10^−06^
	T cell receptor signaling pathway	hsa04660	108	7	2.40*10^−06^
Large AAA-downregulated genes	Metabolic pathways	hsa01100	1104	78	1.05*10^−24^
	Huntington's disease	hsa05016	185	31	3.64*10^−20^
	Oxidative phosphorylation	hsa00190	135	27	8.60*10^−20^
	Alzheimer's disease	hsa05010	169	28	2.45*10^−18^
	Parkinson's disease	hsa05012	133	25	7.70*10^−18^
	Ribosome	hsa03010	88	18	1.32*10^−13^
	Cardiac muscle contraction	hsa04260	79	16	4.25*10^−12^
	Focal adhesion	hsa04510	201	23	8.41*10^−12^
	Vascular smooth muscle contraction	hsa04270	115	17	1.27*10^−10^
	Fatty acid metabolism	hsa00071	42	10	1.70*10^−08^

AAA, abdominal aortic aneurysm; N, number of reference genes in the pathway; n, number of genes differentially expressed in pathway in small or large AAA compared to patients without AAA (Controls); > 2-fold difference, P value < 0.05 calculated with non-parametric Mann-Whitney test and corrected by Benjamini and Hochberg method. P value indicates the corrected significance of enrichment calculated from the hypergeometric test by Benjamini and Hochberg method. Pathways were identified using WebGestalt and were linked to the KEGG (Kyoto Encyclopedia of Genes and Genomes) website http://www.genome.jp/kegg/.

### Differentially expressed genes in small and large AAA

Considering the marked enrichment of immune response categories in small and large AAA, we further examined a number of genes related to the immune response (Table 3). A majority of genes upregulated in small AAA were also upregulated in large AAA. Examples include genes coding for: pro-inflammatory cytokines *IL1B* (5.08-fold, *P* = 0.002 for small AAA; 3.85-fold, *P* = 0.001 for large AAA), *IL6* (6.28-fold, *P* = 0.004 for small AAA; 6.48-fold, *P* = 0.002 for large AAA), and *IL8* (4.49-fold, *P* = 0.005 for small AAA; 3.65-fold, *P* = 0.004 for large AAA); chemokines *CXCL2* (3.35-fold, *P* = 0.004 for small AAA; 2.73-fold, *P* = 0.005 for large AAA), *CXCL13* (2.21-fold, *P* = 0.013 for small AAA; 3.09-fold, *P* = 0.002 for large AAA), *CCL3L3* (3.14-fold, *P* = 0.039 for small AAA; 2.59-fold, *P* = 0.041 for large AAA), and *CCL4L2* (2.48-fold, *P* = 0.021 for small AAA; 2.12-fold, *P* = 0.023 for large AAA); early T-cell and B-cell activation cluster of differentiation (CD) antigens *CD69* (2.67-fold, *P* = 0.039 for small AAA; 3.45-fold, *P* = 0.004 for large AAA) and *CD19* (2.79-fold, *P* = 0.026 for small AAA; 4.45-fold, *P* = 0.002 for large AAA), respectively; and the natural killer (NK) cells associated protein *NKTR* (2.37-fold, *P* = 0.002 for small AAA; 2.66-fold, *P* = 0.001 for large AAA). An important negative regulator of T-cells responses, the *CTLA4* gene, was exclusively upregulated in small AAA (2.01-fold, *P* = 0.018). On the other hand, there was increased expression of genes related to T-cell and B-cell responses exclusively in large AAA, including alpha chain of the T-cell co-receptor *CD8A* (2.24-fold, *P* = 0.012); the *CD79A*/*CD79B* membrane-bound dimer immunoglobulin in B-cells (2.76-fold, *P* = 0.005/3.43-fold, *P* = 0.004); the receptors for Fc fragment of IgG in B-cells *FCRL2* (2.53-fold, *P* = 0.002), *FCRL5* (2.01-fold, *P* = 0.001), and *FCRLA* (3.15-fold, *P* = 0.009). Three genes (*CTLA4*, *NKTR*, and *CD8A*) were selected for further validation with quantitative real-time PCR.

**Table 3:** Examples of differentially expressed genes in small and large AAA with some connection to immunity

Symbol	Name	Small AAA		Large AAA	
		Fold change	Corrected P value	Fold change	Corrected P value
*IL1B*	interleukin 1, beta	5.08	0.002	3.85	0.001
*IL6*	interleukin 6	6.28	0.004	6.48	0.002
*IL8*	interleukin 8	4.49	0.005	3.65	0.004
*CXCL2*	chemokine (C-X-C motif) ligand 2	3.35	0.004	2.73	0.005
*CXCL13*	chemokine (C-X-C motif) ligand 13	2.21	0.013	3.09	0.002
*CCL3L3*	chemokine (C-C motif) ligand 3-like 3	3.14	0.039	2.59	0.041
*CCL4L2*	chemokine (C-C motif) ligand 4-like 2	2.48	0.021	2.12	0.023
*CD69*	CD69 molecule	2.67	0.039	3.45	0.004
*CD19*	CD19 antigen	2.79	0.026	4.45	0.002
*NKTR*	natural killer-tumor recognition sequence	2.37	0.002	2.66	0.001
*CTLA4*	cytotoxic T-lymphocyte-associated protein 4	2.01	0.018	-	-
*CD8A*	CD8 antigen, alpha chain	-	-	2.24	0.012
*CD79A*	CD79A antigen, immunoglobulin-associated alpha	-	-	2.76	0.005
*CD79B*	CD79b molecule, immunoglobulin-associated beta	-	-	3.43	0.004
*FCRL2*	Fc receptor-like 2	-	-	2.53	0.002
*FCRL5*	Fc receptor-like 5	-	-	2.01	0.001
*FCRLA*	Fc receptor-like A	-	-	3.15	0.009

AAA, abdominal aortic aneurysm. Differentially expressed genes were determined by comparison of patients with small or large AAA with patients without AAA (Controls), (> 2-fold change, P value < 0.05 calculated with non-parametric Mann-Whitney test and corrected by Benjamini and Hochberg method).

### Pathways related to the differentially expressed genes in AOD

92% (1,550/1,677) of upregulated genes and 90% (79/88) of downregulated genes in AOD samples were annotated in the KEGG pathways. The pathways enriched in AOD samples were distinct to those identified in AAA samples. In AOD, the most significant KEGG categories representing upregulated genes were metabolic pathways (hsa01100; *P* = 1.49*10^−43^) and oxidative phosphorylation (hsa00190; *P* = 5.68*10^−35^). Downregulated genes primarily fell into the immune response categories such as cytokine-cytokine receptor interaction (hsa04060; *P* = 5.69^−07^) and chemokine signaling pathways (hsa04062; *P* = 8.64*10^−06^) as seen in Table 4.

**Table 4:** Top 10 KEGG pathways enriched in aortic occlusive disease

Category	Pathway	KEGG	N	n	P value
AOD-upregulated genes	Metabolic pathways	hsa01100	1104	147	1.49*10^−43^
	Oxidative phosphorylation	hsa00190	135	49	5.68*10^−35^
	Parkinson's disease	hsa05012	133	46	7.03*10^−32^
	Huntington's disease	hsa05016	185	52	4.11*10^−31^
	Alzheimer's disease	hsa05010	169	48	5.02*10^−29^
	Ribosome	hsa03010	88	35	6.28*10^−27^
	Focal adhesion	hsa04510	201	38	1.90*10^−16^
	Spliceosome	hsa03040	128	29	8.16*10^−15^
	Cardiac muscle contraction	hsa04260	79	21	2.63*10^−12^
	Proteasome	hsa03050	48	17	2.63*10^−12^
AOD-downregulated genes	Cytokine-cytokine receptor interaction	hsa04060	267	8	5.69*10^−07^
	Hematopoietic cell lineage	hsa04640	88	5	5.93*10^−06^
	Chemokine signaling pathway	hsa04062	190	6	8.64*10^−06^
	Regulation of actin cytoskeleton	hsa04810	216	5	2.00*10^−04^
	Graft-versus-host disease	hsa05332	42	3	3.00*10^−04^
	Jak-STAT signaling pathway	hsa04630	155	4	6.00*10^−04^
	Pathogenic Escherichia coli infection	hsa05130	59	3	6.00*10^−04^
	NOD-like receptor signaling pathway	hsa04621	62	3	6.00*10^−04^
	Toll-like receptor signaling pathway	hsa04620	101	3	2.10*10^−04^
	Prion diseases	hsa05020	35	2	3.70*10^−04^

AOD, aortic occlusive disease; N, number of reference genes in the pathway; n, number of genes differentially expressed in pathway in AOD compared to patients without AAA (Controls); > 2-fold difference, P value < 0.05 calculated with non-parametric Mann-Whitney test and corrected by Benjamini and Hochberg method. P value indicates the corrected significance of enrichment calculated from the hypergeometric test by Benjamini and Hochberg method. Pathways were identified using WebGestalt and were linked to the KEGG (Kyoto Encyclopedia of Genes and Genomes) website http://www.genome.jp/kegg/.

### Differentially expressed genes in AOD

Inspection of genes within KEGG metabolic categories revealed upregulation of genes related to mitochondrial biogenesis that included many members of all five mitochondrial respiratory and ATP production complexes (Table 5). We also demonstrated the upregulation of numerous genes involved in glycolysis/gluconeogenesis, fatty acid metabolism, and propanoate metabolism (Table 5). Several complement system genes were overexpressed such as *C1S* (2.08-fold, *P* = 0.044), *CD46* (2.00-fold, *P* = 0.004), *CFH* (2.81-fold, *P* = 0.006), *CFI* (2.02-fold, *P* = 0.004), *C1QBP* (2.10-fold, *P* = 0.003), *C1RL* (2.08-fold, *P* = 0.003), and *C7* (3.46-fold, *P* = 0.037). One important mitochondrial regulatory gene called mitochondrial uncoupling protein 2 (*UCP2*) was downregulated in AOD samples (−3.73-fold, *P* = 0.014), (Table 5). The *UCP2* gene was selected for further validation with quantitative real-time PCR.

**Table 5:** Examples of differentially expressed genes in AOD

Symbol	Name	Function	Fold change	Corrected P value
*NDUFA3*	NADH dehydrogenase (ubiquinone) 1 alpha subcomplex, 3	Complex I	2.71	0.003
*NDUFB3*	NADH dehydrogenase (ubiquinone) 1 beta subcomplex, 3	Complex I	2.53	0.003
*NDUFA7*	NADH dehydrogenase (ubiquinone) 1 alpha subcomplex, 7	Complex I	2.12	0.003
*NDUFB6*	NADH dehydrogenase (ubiquinone) 1 beta subcomplex, 6	Complex I	2.68	0.003
*SDHA*	succinate dehydrogenase complex, subunit A	Complex II	2.00	0.003
*SDHD*	succinate dehydrogenase complex, subunit D	Complex II	2.66	0.003
*UQCRB*	ubiquinol-cytochrome c reductase binding protein	Comples III	2.89	0.003
*UQCRH*	ubiquinol-cytochrome c reductase hinge protein	Comples III	10.76	0.003
*UQCRFS1*	ubiquinol-cytochrome c reductase, Rieske iron-sulfur polypeptide 1	Comples III	2.62	0.003
*COX5B*	cytochrome c oxidase subunit Vb	Complex IV	4.62	0.003
*COX6A1*	cytochrome c oxidase subunit VIa polypeptide 1	Complex IV	2.06	0.008
*COX6B1*	cytochrome c oxidase subunit VIb polypeptide 1	Complex IV	10.45	0.003
*COX6C*	cytochrome c oxidase subunit VIc	Complex IV	3.34	0.003
*COX7C*	cytochrome c oxidase subunit VIIc	Complex V	4.32	0.003
*ATP5C1*	ATP synthase, H+ transporting, mitochondrial F1 complex, gamma polypeptide 1	Complex V	2.22	0.003
*ATP5D*	ATP synthase, H+ transporting, mitochondrial F1 complex, delta subunit	Complex V	2.00	0.005
*ATP5J*	ATP synthase, H+ transporting, mitochondrial F0 complex, subunit F6	Complex V	3.13	0.003
*UCP2*	uncoupling protein 2	Uncoupling	−3.73	0.014
*ALDH1B1*	aldehyde dehydrogenase 1 family, member B1	G/F/P	2.84	0.008
*ALDH2*	aldehyde dehydrogenase 2 family	G/F/P	2.03	0.017
*ALDH3A2*	aldehyde dehydrogenase 3 family, member A2	G/F/P	2.45	0.003
*ALDH9A1*	aldehyde dehydrogenase 9 family, member A1	G/F/P	2.61	0.003
*PDHB*	pyruvate dehydrogenase beta	G	2.64	0.003
*TPI1*	triosephosphate isomerase 1	G	2.23	0.003
*ACADL*	acyl-CoA dehydrogenase, long chain	F	2.04	0.003
*ACAT1*	acetyl-CoA acetyltransferase 1	F	3.81	0.003
*MCEE*	methylmalonyl CoA epimerase	P	2.34	0.003
*MUT*	methylmalonyl CoA mutase	P	2.08	0.003
*C1S*	complement component 1, s subcomponent	Complement	2.08	0.044
*CD46*	CD46 molecule	Complement	2.00	0.004
*CFH*	complement factor H	Complement	2.81	0.006
*CFI*	complement factor I	Complement	2.02	0.004
*C1QBP*	complement component 1, q subcomponent binding protein	Complement	2.10	0.003
*C1RL*	complement component 1, r subcomponent-like	Complement	2.08	0.003
C7	complement component 7	Complement	3.46	0.037

G, Glycolysis/Gluconeogenesis; F, Fatty acid metabolism; P, Propanoate metabolism; Complex I –V, mitochondrial complexes. Differentially expressed genes were determined by comparisons of patients of aortic occlusive disease (AOD) with patients without AAA (Controls), (> 2-fold change, corrected P value < 0.05 calculated with non-parametric Mann-Whitney test and corrected by Benjamini and Hochberg method.

### Real time PCR

**Figure 2:**
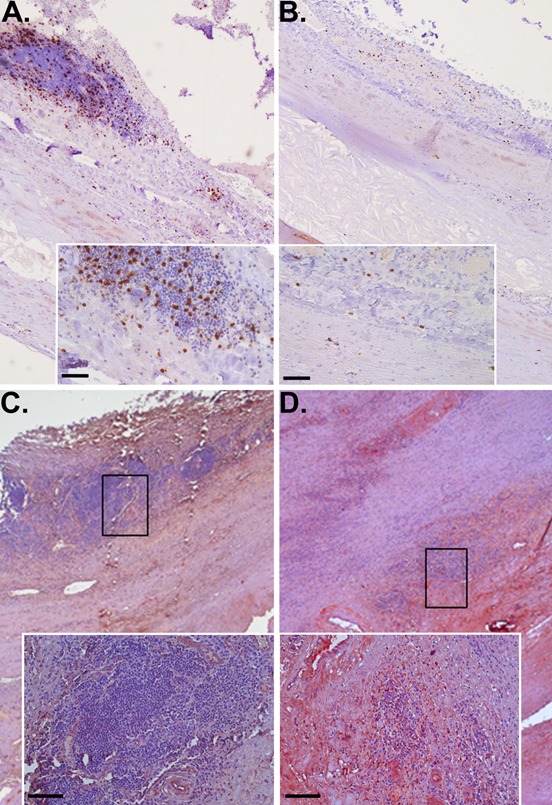
CD8+ lymphocytes and CTLA4 in large versus small AAA Immunohistochemical staining identifying the increased presence of CD8+ T cells (brown stain) in tissue from large AAA (**A**) versus small AAA (**B**) associated with lower detection of CTLA4 (red stain) in large (**C**) compared to small AAA (**D**); scale bar = 50 μm (A & B) & 100 μm (**C** & **D**).

Expression levels of a number of genes were validated in biopsies from the six patients with small AAAs, nine patients with large AAAs, eight patients with AOD and six controls (Table 1). The relative expression of *CTLA4* was significantly increased in small AAA but not large AAA biopsies compared to normal controls (Table 6). Median relative expression of *CTLA4* was also markedly increased in small AAA versus large AAA biopsies (202.161 vs. 65.239, P = 0.041). The relative expression of *CD8A* was significantly increased in large AAA but not small AAA biopsies compared to normal controls (Table 6), with the median relative expression of *CD8A* in large AAA ~2-fold higher and approaching significance compared to small AAA (109.489 *vs* 61.004; *P* = 0.065). Correspondingly, immunohistochemical examination of AAA tissue demonstrated marked presence of CD8-positive T lymphocytes within the adventitia and outer media of a large (82mm) AAA (Figure 2A) with limited immunostaining for CTLA4 (Figure 2C). In contrast there was relatively sparse presence of CD8-positive T cells within tissue from a small (55mm) AAA (Figure 2B) but marked CTLA4 staining around tertiary lymphoid tissue at the media-adventia border (Figure 2D).

**Table 6:** Validation of genes differentially expressed in AAA or AOD by qRT-PCR

Gene	Sample	N	Median	IQR	Fold change	P
*CTLA4*	Small AAA	6	202.161	120.109-376.825	11.12	0.002
	Normal control	6	18.185	10.526-24.477		
	Large AAA	9	65.239	17.268-176.354	3.59	0.065
	Normal control	6	18.185	10.526-24.477		
	Small AAA	6	202.161	120.109-376.825	3.10	0.041
	Large AAA	9	65.239	17.268-176.354		
*CD8A*	Small AAA	6	61.004	51.980-69.598	1.60	0.132
	Normal control	6	38.194	18.075-46.661		
	Large AAA	9	109.487	61.730-176.194	2.87	0.004
	Normal control	6	38.194	18.075-46.661		
	Small AAA	6	61.004	51.980-69.598	−1.79	0.065
	Large AAA	9	109.487	61.730-176.194		
*NKTR*	Small AAA	6	0.651	0.349-0.695	1.66	0.041
	Normal control	6	0.391	0.381-0.404		
	Large AAA	9	1.186	0.771-1.923	3.03	0.015
	Normal control	6	0.391	0.381-0.404		
	Small AAA	6	0.651	0.349-0.695	−1.82	0.040
	Large AAA	9	1.186	0.771-1.923		
*UCP2*	AOD	8	0.729	0.545-0.737	−3.16	0.017
	Normal control	6	2.306	0.909-5.070		

AAA, abdominal aortic aneurysm; AOD, aortic occlusive disease; N, number of samples; Median, median gene expression relative to reference gene (*ACTB*); IQR, interquartile range; P, two-sided P value by Mann-Whitney U test.

Median relative expression of *NKTR* was significantly greater in AAA biopsies compared to normal controls with approximately 2- (*P* = 0.041) and 3- (*P* = 0.015) fold increase for small and large AAAs, respectively (Table 6). Median relative expression of *NKTR* was also markedly increased in large AAA compared to small AAA biopsies (1.186 vs. 0.651, *P* = 0.040).

The relative expression of *UCP2* was markedly decreased in AOD samples compared to normal controls (Table 6). In subjects with AOD the *UCP2* mRNA expression was more than 3-fold lower compared to the control aortic biopsies (0.739 *vs*. 2.306, *P* = 0.017).

## DISCUSSION

Human AAA biopsies have been consistently shown to have intimal atheroma and historically AAAs have been considered to result from atherosclerosis [5, 6]. However the mechanisms involved in AAA development and its relationship with atherosclerosis remain controversial [4]. We determined the global gene expression profiles within biopsies of AAA and AOD using microarrays. The main finding from this study was that the genes differentially expressed in AAA and AOD were almost completely distinct, supporting the concept of separate developmental mechanisms underlying the pathogenesis of each disease [4]. With this in mind, global gene expression profiles associated with AAA and AOD will be further discussed separately.

### AAA

A number of human and animal studies previously suggested a major role of immune responses in AAA pathogenesis [11-15] and the array findings for cytokines/chemokines are in line with previous quantitative data [18]. Our results substantiate those findings, and more importantly, suggest also that small AAAs (≤ 55 mm) and large AAAs (> 55 mm) may have some distinct immunological characteristics. The most notable finding related to immunity was that the key T-cell regulatory gene called cytotoxic T-lymphocyte-associated protein 4 (*CTLA4* or *CD152*) was exclusively found to be upregulated in small AAAs. Moreover, our data suggests excessive infiltration of CD8^+^ T-cells in biopsies from large AAAs evidenced by the exclusive overexpression of the gene coding for the CD8A molecule and increased number of CD8+ T-cells found in these specimens. The upregulation of *CTLA4* and *CD8A* in small AAA and large AAA, respectively, was confirmed by qRT-PCR and led us to hypothesize that downregulation of *CTLA4* may represent a key mechanism in AAA progression which promotes excessive T-cell-driven immune responses in large AAAs. This hypothesis is supported by our finding of marked downregulation of the *CTLA4* mRNA and the protein product in large AAAs compared to small AAAs as determined by qRT-PCR and immunohistochemistry, respectively. CTLA4 is a cell surface molecule that can down-modulate and terminate T-cell adaptive immunity [19]. The CD8A molecule is a constituent of the CD8 antigen found on most cytotoxic T lymphocytes and acts as a co-receptor with the T-cell receptor (TCR). Previous immunohistochemical studies of human AAA have provided evidence that CD8^+^ T-cells infiltrating the aortic wall express cytotoxic mediators such as perforins, which generate membrane damage [20]. Other studies have shown that immunosuppressive agents directed exclusively at T-cells are capable of reducing the expansion of experimental AAA [21] and reduce vascular inflammation and aortic wall content of several inflammatory cell types including cytotoxic T lymphocytes in human AAA [22]. Taken together, the manipulation of CTLA4 levels may represent a novel therapeutic strategy for AAAs but this need examining in other contexts e.g. animal models. The use of CTLA4-based biologic agents such as Abatacept (Orencia), a novel fusion protein of the Fc region of the immunoglobulin IgG1 and CTLA4 designed to down-regulate T-cells activity, is already under investigation as a potential therapy for other immunopathologies such as active rheumatoid arthritis [23-27]. Findings from the present study additionally demonstrate the upregulation of genes expressed by NK cells within large AAAs, such as natural killer cell group 7 sequence (*NKG7*) and natural killer-tumor recognition sequence (*NKTR*) genes; both involved in the TCR-independent innate immune responses. The latter was assessed and validated to be upregulated in large AAAs by qRT-PCR. It is possible that similar mechanisms involving the upregulation of innate immunity occur at some point between the late and end-stage of AAA. Findings suggest marked upregulation of the *NKTR* gene in large AAA compared to small AAA biopsies which is in accord with this hypothesis.

### AOD

Genes differentially expressed in AOD versus control samples were particularly related to the metabolic and oxidative phosphorylation pathways which accounted for ~12% of all upregulated genes. In particular, we determined the excessive transcription of genes involved in mitochondrial biogenesis affecting numerous members of all four mitochondrial respiratory complexes as well as genes responsible for ATP synthesis in the mitochondrial complex V. Other pathways enriched included those related to Parkinson's disease. Genes involved in this category belonging to the mitochondrial complex I were upregulated in AOD, contrasting the actual disruption of the mitochondrial complex I in Parkinson's disease itself [28]. We also demonstrated the upregulation of a range of genes involved in glycolysis, gluconeogenesis, fatty acid metabolism, and propanoate metabolism. Since the number of overlapping genes in these metabolic pathways is considerable, it would be reasonable to suggest that overexpression of these genes may simply represent a metabolic maladaptation to the excessive mitochondrial biogenesis, as members of these bioenergetic pathways are tightly coordinated transcriptionally [29-31]. Upregulation of genes related to glucose and fatty acids metabolism is highly relevant to AOD. The role of an impaired glucose tolerance and fatty acids metabolism in atherosclerosis is long-documented [32, 33]. Current evidence suggests that the ataxia telangiectasia mutated (ATM)/mammalian target of rapamycin (mTOR) signaling axis may play an important role in suppression of macrophage transformation into foam cells, thus limiting the formation of atherosclerotic plaques [34]. Interestingly, the old malaria drug Chloroquine (Aralen), a lysosomotropic agent that activates ATM signaling, was found to be capable of decreasing atherosclerosis and improving metabolic phenotype in mice [35]. The hypothesis that Chloroquine may also reduce atherosclerosis in humans is currently under investigation [36]. The mechanisms responsible for upregulation of genes involved in mitochondrial biogenesis are not evident in the current study; however, we did identify downregulation of the *UCP2* gene in AOD samples. The UCP2 protein belongs to a group of mitochondrial transporters that create proton leaks across the inner mitochondrial membrane, thus uncoupling respiration from ATP synthesis [37]. Previous studies provide evidence that uncoupling by UCP2 downregulates the production of reactive oxygen species (ROS) in endothelial and smooth muscle cells [38-42], whereas the UCP2 deficiency is associated with enhanced ROS production in the endothelium of the aorta [43]. Excessive ROS production is known to contribute to the oxidative damage of mitochondria that needs to be repaired to maintain intact cellular mitochondrial content [44]. This process is accomplished via mitophagy (autophagy), a selective elimination of malfunctioning mitochondria, which must be balanced by mitochondrial biogenesis to meet tissue energy requirements [45]. It is possible that UCP2 deficiency may, in fact, indirectly lead to increased mitochondrial biogenesis, explaining the upregulation of genes related to the metabolic and oxidative phosphorylation pathways in AOD. Pathways related to inflammation were less evident amongst differentially expressed genes in AOD samples. We did demonstrate the upregulation of a number of complement regulatory and effectors genes, such as complement regulatory protein *CD46*, complement factor H (*CFH*), and complement factor I (*CFI*) suggesting potential involvement of the classical complement activation pathway in AOD. The overexpression of the complement component 7 (*C7*) gene, a constituent of the membrane attack complex (MAC), may indicate the culmination of the alternative complement activation pathway in AOD. Findings are in line with previous studies that have suggested an important role for the complement system in mediating tissue injury and atherosclerosis after oxidative stress [46, 47].

### Limitation of the study

The current study has a number of limitations. The available sample size was relatively small especially for AOD although larger than any previously published microarrays using human aortic biopsies. In view of this limitation we sought to validate important findings in further groups of AAA and AOD biopsies. The assessment of independent samples helps to minimize the possibility that selection biases adversely affect the generalizability of the findings. Furthermore, we were able to obtain the aortic biopsies from advanced stage disease including AAAs measuring at least 50mm and patients needing aortic bypass for peripheral ischemia. Our findings therefore are difficult to relate to early stage vascular disease. The majority of available AAA and AOD biopsies were obtained from men limiting the relevance of our findings to AAA and AOD in women. Finally, the AOD patients were either younger (discovery group) or older (validation group) than their respective controls. In both particular comparisons of cases with controls, however, an important gene regulating mitochondrial biogenesis was found to be downregulated in AOD, suggesting that our findings are descriptive of the disease rather than age differences between cases and controls.

In conclusion, the current results demonstrate distinct gene expression profiles of AAA and AOD. The AAA transcriptome highlights an important mechanism controlling immune responses as opposed to the mitochondrial biogenesis pathways being predominantly associated with AOD pathology.

## MATERIALS AND METHODS

A detailed description of all methods is presented in the online data supplement.

### Patients

Full thickness abdominal aortic specimens were obtained from 49 patients undergoing open surgery to treat AAA, 9 patients with AOD undergoing surgery to treat chronic lower limb ischemia, and 10 control individuals (discovery group, Table 1). Maximum aortic diameter was 50-55mm (defined as small AAA) and 56-120mm (defined as large AAA) in 20 and 29 AAA subjects, respectively. Control full thickness abdominal aortic samples were obtained during kidney transplant (all from heart-beating, brain-dead donors). The study was approved by the ethics committees of the Townsville Hospital, James Cook University, Royal Brisbane and Women's Hospital, Medical Faculty at the Technical University Dresden, and Leiden University Medical Center and the protocol conformed to ethical guidelines of Declaration of Helsinki. All patients gave written informed consent.

### Microarrays

Microarrays were performed using the Illumina HumanHT-12v4 Expression BeadChip^®^ platform as previously described [16]. The microarray data can be obtained at the Gene Expression Omnibus (GEO) database repository (GSE57691; http://www.ncbi.nlm.nih.gov/geo/info/linking.html).

### Analysis of array data

The raw data matrix extracted from BeadStudio was uploaded into the GeneSpring GX version 11.5.1 (Agilent Technologies Pty Ltd) software for downstream analysis as previously described [16]. We sought to identify genes with a 2-fold differential expression within the aortas of patients with small AAA, large AAA or AOD compared to donor abdominal aortas based on corrected p value < 0.05 by the Benjamini Hochberg false discovery rate (FDR) method and determined by non-parametric Mann–Whitney U test. Genes showing ≥ 2-fold differences in expression between groups were considered to be significantly differentially expressed.

### Quantitative real-time reverse transcription polymerase chain reaction (qRT-PCR)

Using total RNA obtained from a further 29 patients with small AAAs (*n* = 6), large AAAs (*n* = 9) and AOD (*n* = 8), and another 6 organ donor biopsies (validation group, Table 1) we assessed the validity of microarrays findings using qRT-PCR as described previously [16, 17]. Three genes associated with AAA (*CTLA4*, cytotoxic T-lymphocyte-associated protein 4; *NKTR*, natural killer cell triggering receptor; *CD8A*, CD8A molecule), and the uncoupling protein 2 gene (*UCP2*) associated with AOD were chosen for further assessment. Mann–Whitney U test was performed to identify differences in expression levels of selected genes between small AAA, or large AAA, or AOD and control biopsies. Data are reported as median and interquartile range. All computations were performed using the SPSS statistical package v.17.0.2. Statistical significance was defined at the conventional 5% level.

### Immunohistochemistry

CD8 and CTLA4 proteins were immunohistochemically detected on 5-μm thick paraffin sections of AAA tissue taken from the maximally dilated aneurysm body as previously described [17].

## SUPPLEMENTARY MATERIAL TABLES






